# The Effects of Dynamic Work Environments on Entrepreneurs’ Humble Leader Behaviors: Based on Uncertainty Reduction Theory

**DOI:** 10.3389/fpsyg.2019.02732

**Published:** 2019-12-19

**Authors:** Xiao Deng, Bo Gao, Guozheng Li

**Affiliations:** ^1^Business School, China University of Political Science and Law, Beijing, China; ^2^School of Government and Public Affairs, Communication University of China, Beijing, China; ^3^School of Economics and Management, Beijing University of Technology, Beijing, China

**Keywords:** dynamic work environments, humble leader behaviors, feedback-seeking behavior, uncertainty reduction theory, intolerance of uncertainty

## Abstract

Although it is widely acknowledged that the environments faced by entrepreneurs now are more dynamic than ever, little is known about the effect of dynamic work environments on entrepreneurs’ leader behaviors. Based on the uncertainty reduction theory and the data from 197 entrepreneurs and their subordinates, this research found a positive relationship between dynamic work environments and entrepreneurs’ humble leader behaviors. Moreover, this positive relationship can be mediated by entrepreneurs’ feedback-seeking behavior. And the relationship between dynamic work environments and entrepreneurs’ humble leader behaviors (*via* feedback-seeking) can be moderated by entrepreneurs’ intolerance of uncertainty. The contributions and implications of this study are discussed.

## Introduction

Owing to the imperfect and unbalanced legal and financial supports, relatively unpredictable market demand, rapidly upgrading technologies, and fierce competition (Li and Atuahene-Gima, 2001), entrepreneurial teams, compared to established organizations, confront a more dynamic environment (Brouwer, 2000). Exploring how dynamic environments can affect entrepreneurial teams is very important to know more about entrepreneurial teams and can be very helpful for entrepreneurial teams to seize such kinds of opportunities and avoid many traps in the current dynamic environments (Stinchcombe, 1965). Previous research in this field remarkably focuses on how dynamic environments influence the different strategies entrepreneurial teams adopt. However, few researchers paid attention to the influence of dynamic environments on entrepreneur behaviors. As a matter of fact, entrepreneur behaviors have been considered to be a vital part to the performance of entrepreneurial teams under different situations (Chandler and Hanks, 1994; Obschonka et al., 2018). Thus, in this paper, we will try to fill this research gap by exploring how dynamic environments affect entrepreneur behaviors.

According to traditional leadership convention, leaders are considered to be proactive and can react to the environments by taking different leadership behaviors to improve effectiveness. The likelihood of making mistakes caused by the narcissism and hubris of leaders would increase in dynamic environments (Pawar and Eastman, 1997; Shamir and Howell, 1999; Rogoza et al., 2018). More and more researchers claim that the current turbulent environment may push leaders to engage in more “bottom–up” leadership styles (Kerfoot, 1998; Weick, 2001; Vera and Rodriguez-Lopez, 2004; Morris et al., 2005), among which humble leader behaviors have the most obvious representativeness. The situation that leaders acting as being humble is beneficial for collecting additional information and making use of collective intelligence in dynamic environments (e.g., Wang et al., 2018). Therefore, we propose that dynamic environments may contribute entrepreneurs to exhibit more humble leader behaviors.

To explain the relationship in detail, we applied the uncertainty reduction theory as an overarching theory, which asserts that uncertainty can motivate people to exhibit more specific behaviors to reduce uncertainty (Knobloch, 1975, 2005). Meanwhile, feedback-seeking has been shown as an effective way to reduce the uncertainty brought by dynamic external environments (Morrison, 2002; Tuckey et al., 2002; Shen et al., 2019), and it enables people to understand their strengths and weaknesses better, to acknowledge others’ thoughts and understand them well, and receive different information and ideas from all kinds of levels in this field. All these factors contribute to humble leader behaviors (Tangney, 2000; Exline and Geyer, 2004; Owens, 2009; Owens et al., 2013). Thus, we propose, in highly dynamic work environments, to reduce the potential hazard of uncertainty brought by environmental dynamics, leaders can react by seeking feedbacks, which would exhibit more humble leader behaviors.

**FIGURE 1:**
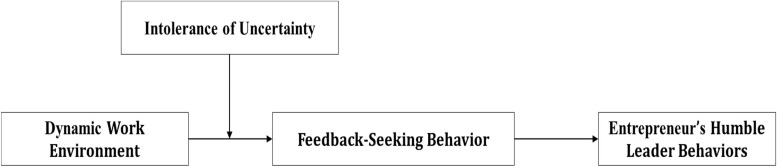
Research framework.

Further more, besides contextual factors, leaders’ personal character differences are also important to their choices of leadership behaviors (Bono and Judge, 2004). Therefore, we take leaders’ traits into consideration as a moderator of the relationship between dynamic work environments and entrepreneurs’ humble leader behaviors. The trait of intolerance of uncertainty can affect individuals’ cognition and emotion toward dynamic environments—individuals with high intolerance of uncertainty may tend to react in a negative way to dynamic environments (e.g., Lauriola et al., 2018). Thus, entrepreneurs with high intolerance of uncertainty may be too anxious to react to the dynamic work environment by applying feedback seeking and humble leader behaviors. We propose entrepreneurs’ intolerance of uncertainty can be a moderator and weaken the relationship between dynamic environments and entrepreneurs’ humble leader behaviors. The whole research framework model is shown in Figure 1.

Our theoretical constructions and empirical findings come to get a reach of three significant contributions. First, although many researchers have paid attention to the influence of dynamic environments on entrepreneurial teams, few researchers focused on the influence on entrepreneurs’ behaviors. Our study tries to fill this gap by examining the impact of dynamic environments on entrepreneurs’ humble leader behavior. Second, by revealing dynamic work environments that can be the antecedents of humble leader behaviors, our research broadens the knowledge of leader humble behaviors and contributes to the understanding of leadership. Thirdly, our work reveals the whole mechanism of how and when dynamic environments can affect entrepreneurs’ humble leader behaviors. We propose feedback-seeking behavior as a mediator and leaders’ intolerance of uncertainty as a moderator. By revealing the influence mechanism of dynamic environments on entrepreneurs’ humble leader behaviors and boundary conditions, this study explains the current phenomenon of emergence of leader’s humility and provides a better understanding of the influence of dynamic work environments.

### Theoretical Background

#### Uncertainty Reduction Theory

Uncertainty could result in undesired effects or significant losses (Antunes and Gonzalez, 2015). As humans, we try to avoid uncertainty. The uncertainty reduction theory (Knobloch, 1975, 2005) is one of the few psychological theories that are specifically researched in the initial interaction between individuals, particularly before communication begins. This theory emphasizes the notion that uncertainty reduction could be one of the leading motives behind some behaviors. Through specific behaviors, more information could be gained and verified in order to reduce the uncertainty brought by the unknown (Tannert et al., 2007). When confronted with uncertainty, people tend to be motivated to exhibit these specific behaviors to decrease uncertainty. This theory has been already applied in organizational studies. For instance, based on this theory, Ashford and Cummings (1985), by studying empirical data, proved that when employees experience a great deal of uncertainty (as reflected by their role ambiguity and contingency uncertainty), to obtain an accurate self -evaluation and confirm one’s perceptions, they tend to seek more feedback.

Dynamism is a key characteristic of an environment that indicates a degree of rapid, unpredictable, and turbulent change (Shamir and Howell, 1999; Eisenhardt and Martin, 2000). In highly dynamic work situations, “there is rapid and discontinuous change in demand, competitors, technology and/or regulation, such that information is often inaccurate, unavailable, or obsolete” (Eisenhardt and Bourgeois, 1988). In such environments, entrepreneurs may feel more uncertain due to the lack of the latest information, uncertainty of the outcomes of specific inputs, and the possible lagged or conflicting feedback from environments (Lawrence and Lorsch, 1967). In entrepreneurial teams in China, owing to their relatively high unpredictable market demand and consumers’ tastes, fierce competition, and sudden changes in legal, political, and economic constraints (Li and Atuahene-Gima, 2001), highly dynamic environments are very common. Moreover, the work environments entrepreneurs face now are more dynamic than ever (Senge, 1990). Under these circumstances, entrepreneurs may perceive very high levels of uncertainty about gaining vital and new information and the existence of unclear outcomes of decisions. According to the uncertainty reduction theory, the more dynamic work environments are, the more specific behaviors entrepreneurs will exhibit to reduce uncertainty.

#### Dynamic Work Environments and Humble Leader Behaviors

The term humility comes from the Latin words *humus*, meaning “earth,” and *humi*, “on the ground” (as per the Online Etymology Dictionary, accessed in 2010). Thus, humble leader behaviors literally means “leading from the ground” or “bottom–up leadership” (Owens and Hekman, 2012). In the past decades, with increased negative results caused by “heroic leaders” (Bowles, 1997; Collins, 2001; Raelin, 2003), leadership thinkers have come to increasingly focus on the importance of humility in the context of leadership. The perspectives of servant leadership (Greenleaf and Spears, 2002), level 5 leadership (Collins, 2001), and participative leadership (Kim, 2002) specifically highlight the virtues of humble behavior as being critical for leadership effectiveness (cf. Weick, 2001). Empirically, humble leader behaviors have been proven to result in positive outcomes for their teams and subordinates, such as positively fostering deviant behavior (i.e., exceptional performance and prosaically behavior) in the workplace (Cameron and Caza, 2004; Zhou and Wu, 2018; Zhu et al., 2019). With the development of the literature on humble leadership, three features have come to be seen as essential in the definition of humble leader behaviors: viewing themselves more objectively, viewing others more appreciatively, and being open to new information and ideas (Templeton, 1997; Tangney, 2000; Exline and Geyer, 2004; Owens et al., 2013). That is to say, leaders exhibiting humble leader behaviors tend to acknowledge personal limitations, spotlight followers’ strengths and contributions, and welcome new information and ideas (Owens and Hekman, 2012).

In the last 10 years, leadership thinkers have increasingly come to focus on the importance of humility in the level of leadership. Especially as organizational environments become more dynamic, uncertain and unpredictable, researchers think it becomes increasingly difficult for every leader to “figure it all out at the top” (Senge, 1990, p. 7). Thus, scholars and practitioners have argued that leaders with humility are necessary (Kerfoot, 1998; Vera and Rodriguez-Lopez, 2004; Morris et al., 2005). Concurring with these arguments, we think, entrepreneurs, when faced with dynamic work environments, would exhibit more humble leader behaviors for the following reasons.

First, when the environment is extremely dynamic, meeting with the latest requirements and the changes of environments may affect the survival and the success of entrepreneurial teams a lot (Hmieleski and Ensley, 2007; Diakanastasi and Karagiannaki, 2016; Smithson et al., 2019). From the perspective of individuals’ attention, compared to those in stable environments, entrepreneurs in dynamic environments would pay more attention to the environments. When focusing more on the environments rather than oneself, the frame of references of entrepreneurs will change. Entrepreneurs may have a sense that they are only parts of something larger than themselves (Piff et al., 2015). They tend to notice the power of the environments and limitations of themselves, and do self-evaluating with considering the background of the whole picture (Aron et al., 1992). As a result, entrepreneurs will avoid cognitive biases brought by inflated egos and undertake a more accurate self-evaluation.

Second, as it becomes increasingly difficult for every one to “figure it all out at the top” in dynamic environments (Senge, 1990, p. 7), entrepreneurs start to need the help from others or rely on the whole teams’ contributions to collect enough information and analyze the environments to get the right managerial decisions (e.g., Qian et al., 2018). To do so, entrepreneurs should show more respect and appreciations to their employees, value their inputs, more proactively seek feedbacks and help from their employees. Only in these ways, entrepreneurs can get more knowledge and information about the requirements of dynamic environments and formulate better reactions to dynamic environments. Thus, to reduce the uncertainty brought by speedy changes and integrated information, entrepreneurs would show more respect and appreciation to their employees to get more help from them and make full use of collective intelligence.

Third, being involved in dynamic environments will make individuals exposed to ample new information and changes. To achieve goals under these dynamic environments, individuals have to get used to new things, accept new things, and take quick reactions to new things. Thus, entrepreneurs in dynamic environments would get more accustomed to new things so as to be better competitive and seize all kinds of better opportunities in such situations; they will even appreciate and welcome new things.

To reduce uncertainty and achieve effectiveness in dynamic environments, entrepreneurs who perceive high levels of work environment dynamism are more likely to have accurate self-evaluation, value others’ work and thoughts, and welcome new and diverse information. All of these are the features of humble leader behaviors. Here, we proposed the following:

**Hypothesis 1:** Dynamic work environments have a positive relationship with entrepreneur’s humble leader behaviors.

#### The Mediation Effect of Feedback-Seeking Behavior

Feedback seeking refers to individuals’ proactive desire to obtain evaluation information on their work performance (Ashford and Tsui, 1991; Porath and Bateman, 2006). In organizations, individuals seek feedbacks either by directly asking others for feedbacks (inquiry) or by observing their environments and others for cues that might serve as feedback information (monitoring). By seeking feedback, individuals can better assess their capabilities (Williams and Johnson, 2000), adjust their goal-directed behaviors (Morrison and Weldon, 1990), “learn the ropes” of a new job (Morrison, 1993), and improve their overall work performances (Renn and Fedor, 2001).

Scholars have proposed that the feelings of uncertainty of individuals are the primary determinant in seeking out feedback (Ashford and Cummings, 1983; Morrison, 2002; Ashford et al., 2003; Morrison et al., 2004). This is in line with the uncertainty reduction theory, which predicts that people have an aversion to uncertainty and gather information to reduce such feelings of uncertainty. Thus, we consider that dynamic work environments will increase entrepreneurs’ feelings of uncertainty, and according to the uncertainty reduction theory, leaders will exhibit more feedback-seeking behavior.

Further, we think feedback seeking is important for leaders to exhibit more humble behaviors. Feedback is a typical information that details how others perceive and evaluate individuals’ behaviors and other related things (Powers, 1973; Ilgen et al., 1979; Ashford and Cummings, 1983). First, leaders seek feedback to gain an understanding of how others view them and obtain diagnostic information about themselves. Feedbacks include both the advantages and disadvantages of leaders. The information and evaluation they obtain allows them to have a more accurate evaluation of their self (Sedikides and Strube, 1997). Second, seeking feedback intentionally enables a more widely discussion with subordinates. Entrepreneurs can collect more information about other team members, such as ideas, thoughts, and potential. This results in the improved understanding of subordinates and appreciation of subordinates’ work and contributions. Third, feedback exposes entrepreneurs to different opinions and alternative suggestions that could better improve the final decisions and leaders themselves. Thus, seeking frequent feedback can enable leaders to accept new information and ideas more openly. Hence, feedback seeking can boost the three important features of humble leader behaviors.

Concurring with the uncertainty reduction theory, when entrepreneurs perceive their work environments to be dynamic, they will be motivated to seek feedback by the desire to reduce uncertainty. The process of feedback-seeking allows entrepreneurs to secure accurate knowledge about themselves, know more about others and appreciate others, and be accustomed to different and new information as well as ideas. This results in entrepreneurs exhibiting more humble leader behaviors. To summarize, we claim that feedback-seeking behavior can mediate the relationship between dynamic work environments and humble leader behaviors of entrepreneurs. We propose the hypothesis 2 as follows:

**Hypothesis 2:** Entrepreneur’s feedback-seeking behavior mediates the positive relationship between dynamic work environments and entrepreneur’s humble leader behaviors.

#### The Moderation Effect of Intolerance of Uncertainty

Previous research has emphasized the importance of considering contextual factors and personal factors together when considering the antecedents of leadership. Setting aside the contextual factors, leaders’ characteristics can not only affect their leadership behaviors but also affect how they react to specific contexts (Pawar and Eastman, 1997; Shamir and Howell, 1999; Bono and Judge, 2004).

Intolerance of uncertainty is a trait defined as “the tendency to react negatively on an emotional, cognitive and behavioral level to uncertain situations and events” (Dugas et al., 2004). It is a relevant and stable trait that can affect people’s reaction to uncertainty largely. Individuals with high intolerance of uncertainty tend to have negative reactions to uncertain situations, such as “uncertainty keeps me from sleeping soundly” and “when I am uncertain I can’t go forward” (Buhr and Dugas, 2002). Berenbaum et al. (2008) stated that when people were faced with uncertainty, those with high intolerance of uncertainty have the tendency to become paralyzed by uncertainty. They respond to uncertainty with distress and are unable to take actions to effectively reduce uncertainty. As in the dynamic work environments, entrepreneurs are faced with hardships when processing task related information, instant feedback, and controllable results (Knight, 1921; Luce and Raiffa, 1957; Lawrence and Lorsch, 1967), entrepreneurs’ perception of uncertainty can be high in such environments. For entrepreneurs with high intolerance of uncertainty, high dynamic work environments will swallow them by arousing their negative attitudes and emotions. Thus, they cannot perform uncertainty-reducing behaviors, which include feedback-seeking and humble leader behaviors. Only those entrepreneurs with low intolerance of uncertainty can react to dynamic environments positively by taking actions like feedback-seeking and exhibiting humble behaviors.

Therefore, we suggest that entrepreneurs’ intolerance of uncertainty should moderate the mediation relationship between dynamic work environments, feedback-seeking, and humble leader behaviors in such a way that when entrepreneurs’ intolerance of uncertainty is in a high level, the relationship between dynamic work environments and humble leader behaviors (*via* feedback-seeking behavior) will be weakened.

**Hypothesis 3:** Entrepreneurs’ intolerance of uncertainty moderates the relationship between dynamic work environments and the humble leader behaviors of entrepreneurs (*via* feedback-seeking behavior).

## Materials and Methods

### Sample and Procedures

This study invited 194 entrepreneurs and their subordinates from two business incubators in Beijing of China. One of the incubators is only for the entrepreneurial teams in the industry of Internet education. And the other one is for the entrepreneurial teams in the high-tech industry and provides special entrepreneurial training for entrepreneurs. Entrepreneurial teams from both incubators are involved in dynamic environments. We briefed the participants about the purpose of the study and explained the procedures for completing online surveys. Additionally, we emphasized that others would not have access to their responses or any identifiable information. To better protect the confidentiality of the participants, we assigned random identification numbers to each participant so that we could later match entrepreneur and team member responses respectively.

To prevent common method bias, we collected data in three waves, with intervals of 1–2 weeks. In the first wave, dynamic work environments and intolerance of uncertainty were measured. In the second wave, the entrepreneurs were asked to rate their feedback-seeking behavior. And team members were asked to measure the extent of the humble leader behaviors of their leaders in the third wave. In total, we asked 250 entrepreneurs and their team members to participate in our research. After 3 surveys, we have 194 entrepreneur and team member effective responses that can be matched. The data is shown in Supplementary Table S1. Among the 194 entrepreneurs, 136 (70.1%) were males and only 58 were female. Most of them had graduate degrees since entrepreneurs in the industry of Internet education usually have good education background. And their average work tenure is 29.39 months.

### Measures

According to Brislin (1970) translation-back-translation procedure, all the questionnaires were translated into and printed in Chinese. All the surveys were rated utilizing a seven-point Likert scale ranging from 1 (strongly disagree) to 7 (strongly agree).

#### Dynamic Work Environments

We used entrepreneurs’ perception of the work environment to measure dynamic work environments by the three-item scale from De Hoogh et al. (2005). One typical item was “your work environment is dynamic.” The Cronbach’s alpha was 0.93.

#### Feedback-Seeking Behavior

We assessed feedback-seeking behavior using a seven-item scale adapted from Ashford (1986). Typical items include “I will seek feedbacks about my performance.” The Cronbach’s alpha was 0.96.

#### Humble Leader Behaviors

The nine-item scale from Owens et al. (2013) was used to measure humble leader behavior. One typical item was “My leader is willing to learn from others.” The Cronbach’s alpha was 0.92.

#### Intolerance of Uncertainty

Intolerance of uncertainty was measured with a 12-item scale from Carleton et al. (2007). Typical items include “Uncertainty makes me uneasy, anxious, or stressed.” The Cronbach’s alpha was 0.94.

#### Control Variables

We controlled four variables that have shown to be related to entrepreneur’s leader humble behaviors—entrepreneur’s work tenure, entrepreneur’s education, entrepreneur’s sex, and entrepreneur’s humility personality. We adopted the six-item scale from the HEXACO personality inventory (Lee and Ashton, 2004) to measure entrepreneur’s humility personality. The Cronbach’s alpha was 0.89.

## Results

Before testing the hypotheses, we did CFA analyses to test the discriminant validity of variables. We used Mplus7.4 to do the CFA analyses. During the process, we constructed six model with different factors, and compare the fit indexes of different models. The fit indexes we used include χ^2^, TLI (NNFI), CFI, RMSEA, and SRMR (Ployhart and Oswald, 2004; Brown, 2006). The results are shown in the Table 1. It shows that five factor model is better than others, indicating good discriminate validity of variables.

**TABLE 1:** Results for CFA analyses.

**Model**	**χ^2^**	**df**	**RMSEA (90%CI)**	**TLI**	**CFI**	**SRMR**
5 factors	1367.11^∗∗∗^	619	0.079[0.073,0.085]	0.89	0.87	0.04
4 factors (Intolerance of uncertainty + Entrepreneur humility personality)	2975.18^∗∗∗^	623	0.140[0.134,0.145]	0.63	0.61	0.19
4 factors (Dynamic work environments + Feekback seeking behavior)	1807.63^∗∗∗^	623	0.099[0.094,0.104]	0.82	0.8	0.06
3 factors (Dynamic work environments + Intolerance of uncertainty + Entrepreneur humility personality)	3623.16^∗∗∗^	626	0.157[0.152,0.162]	0.53	0.5	0.22
2 factors (Dynamic work environments + Feekback seeking behavior + Intolerance of uncertainty + Entrepreneur humility personality)	4050.76^∗∗∗^	628	0.168[0.163,0.173]	0.46	0.43	0.22
1 factor	4858.60^∗∗∗^	629	0.186[0.181,0.191]	0.34	0.3	0.23

**N* = 194. ^∗^*p* < 0.05, ^∗∗^*p* < 0.01, ^∗∗∗^*p* < 0.001.*

Since all our variables are on the same level, all study hypotheses were tested using one level modeling. Means, Standard deviations, and correlation coefficients for all measures are shown in Table 2. Dynamic work environments is significantly correlated with feedback-seeking behavior (γ = 0.401, *p* < 0.01) and humble leader behaviors (γ = 0.208, *p* < 0.01).

**TABLE 2:** Descriptive statistics and correlations for study variables.

	***M***	***SD***	**1**	**2**	**3**	**4**	**5**	**6**	**7**
(1) Dynamic work environments	4.68	1.20							
(2) Humble leader behaviors	5.56	1.00	0.208^∗∗^						
(3) Feekback seeking behavior	5.37	1.10	0.401^∗∗^	0.498^∗∗^					
(4) Intolerance of uncertainty	4.57	1.10	–0.050	0.114	–0.065				
(5) Entrepreneur sex	0.70	0.46	0.042	0.055	–0.064	0.173^∗^			
(6) Entrepreneur education	3.02	0.58	0.077	–0.081	–0.018	0.079	0.023		
(7) Entrepreneur tenure	29.39	36.01	0.087	–0.097	–0.093	0.226^∗∗^	0.041	0.197^∗∗^	
(8) Entrepreneur humility personality	5.84	1.07	–0.125	0.391^∗∗^	0.094	–0.026	0.026	−0.212^∗^	−0.144^∗^

**N* = 194. ^∗^*p* < 0.05, ^∗∗^*p* < 0.01, ^∗∗∗^*p* < 0.001. For sex, 0 = “female,” 1 = “male.” For education, 1 = “under bachelor,” 2 = “bachelor,” 3 = “master,” 4 = “doctor and above.” For tenure, n = month.*

We then ran regression to test the hypotheses, as summarized in Table 3. The Model 5 showed a significant main effect of dynamic work environments on humble leader behaviors (β = 0.219, *p* < 0.001). Hypothesis 1 was supported.

**TABLE 3:** Results of hierarchical regression analyses.

	**Feedback-seeking behavior**			**Humble leader behaviors**		
	**1**	**2**	**3**	**4**	**5**	**6**
Entrepreneur sex	–0.152	–0.195	–0.225	0.101	0.077	0.075
Entrepreneur education	0.035	0.001	–0.005	0.016	–0.003	–0.017
Entrepreneur tenure	–0.002	–0.003	–0.003	–0.016	–0.002	–0.001
Entrepreneur humility personality	0.090	0.138^∗^	0.128	0.358^∗∗∗^	0.384^∗∗∗^	0.328^∗∗∗^
Dynamic work environments		0.393^∗∗∗^	1.259^∗∗∗^		0.219^∗∗∗^	0.545^∗^
Intolerance of uncertainty			0.857^∗∗∗^			0.601^∗∗^
Dynamic work environments^∗^ Intolerance of uncertainty			–0.186^∗∗∗^			−0.099^∗^
Feedback-seeking behavior						0.367^∗∗∗^
*R*^2^	0.020	0.201	0.247	0.157	0.225	0.416
Δ*R*^2^		0.181	0.046		0.068	0.191

**N* = 194. ^∗^*p* < 0.05, ^∗∗^*p* < 0.01, ^∗∗∗^*p* < 0.001.*

To test Hypotheses 2 and 3, we utilized the methods of Hayes (2013) to test for conditional indirect effects. This method involves a bias-corrected bootstrapping procedure (5,000 resamples) to compute indirect effects because traditional methods (e.g., Baron and Kenny, 1986) in testing mediation are generally low in power (Fritz and MacKinnon, 2007). The results show that the relationship between dynamic work environments and humble leader behaviors was significantly mediated by feedback-seeking behavior (*R*^2^ = 0.448, *p* < 0.001). Moreover, the indirect effect of dynamic work environments on humble leader behaviors is significant (β = 0.156), and the 95% bias-corrected confidence interval around the bootstrapped indirect effect did not contain zero (bias-corrected CI = [0.096,0.247]). So, Hypothesis 2 is supported.

**FIGURE 2:**
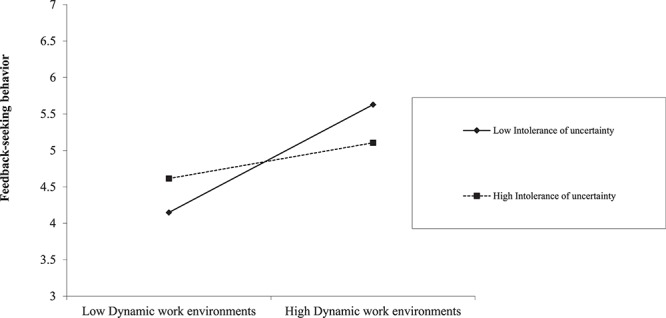
The moderating effect of intolerance of uncertainty on the relationship between Dynamic work environments and feedback seeking behavior.

**FIGURE 3:**
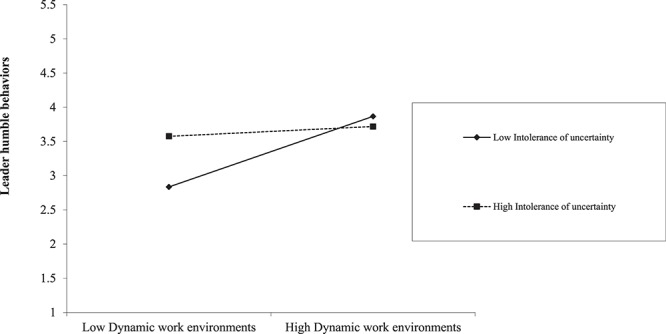
The moderating effect of intolerance of uncertainty on the relationship between Dynamic work environments and leader humble behaviors.

As shown in Table 4, the indirect effect of dynamic work environments on humble leader behaviors was significant and positive when the leader’s intolerance of uncertainty was low (indirect effect = 0.24, *SE* = 0.07, CI [0.12,0.38]), at mean levels (indirect effect = 0.16, *SE* = 0.04, CI [0.10,0.24]), and high (indirect effect = 0.08, *SE* = 0.04, CI [0.01,0.16]. The index of moderated mediation was significant, again indicating a meaningful role of intolerance of uncertainty in the effects of dynamic work environments (indirect effect = −0.07, *SE* = 0.04, CI [−0.16, −0.02]). So, Hypothesis 3 is supported. The moderation effect of intolerance of uncertainty on the relationship between dynamic work environments and feedback seeking behavior is shown in Figure 2. When the intolerance of uncertainty is high, the slope of the relationship between dynamic environments and feedback seeking behavior is milder. And the moderation effect of intolerance of uncertainty on the relationship between dynamic work environments and humble leader behaviors is shown in Figure 3. When the intolerance of uncertainty is high, the slope of the relationship between dynamic environments and leader humble behaviors is milder.

**TABLE 4:** Bootstrapping results for test of conditional indirect effects and index of moderated mediation.

**Dependent variable**	**Level of moderator**	**Effect size**	**Bootstrapped SE**	**Bootstrapped 95% CI**
Leader humble behavior	−1 SD	0.24	0.07	[0.12,0.38]
	Mean	0.16	0.04	[0.10,0.24]
	+1 SD	0.08	0.04	[0.01,0.16]
	Index of moderated mediation	–0.07	0.04	[−0.16, −0.02]

**N* = 194. CI, confidence interval. Results are based on 5,000 bootstrapped samples.*

## Discussion

Using uncertainty reduction theory as an overarching theory, this research found that there exists a positive relationship between dynamic work environments and the humble leader behaviors of entrepreneurs. It further proved a moderated mediation model between dynamic work environments and humble leader behavior, where feedback-seeking behavior was identified as the mediator and entrepreneurs’ intolerance of uncertainty as the moderator. This research revealed the link between dynamic work environments and humble leader behaviors of entrepreneurs, as well as its mechanism and boundary condition.

### Theoretical Contributions

Our findings illustrate several theoretical implications. First, our research has emphasized and proved the influence of dynamic work environments on entrepreneurs’ leadership behaviors. Dynamism is one of the key characteristics of the environments that entrepreneurial teams are confronted with (Senge, 1990; De Hoogh et al., 2005). Reacting well to dynamic environments is important for entrepreneurial teams’ survival and success (e.g., Hmieleski and Ensley, 2007; Diakanastasi and Karagiannaki, 2016). Although previous works have explored how entrepreneurial teams react to dynamic environments, few has focused on entrepreneurs’ reactions to dynamic environments. Examining the effects of dynamic environments on entrepreneurs can not only fill this gap, but also enrich our knowledge about the relationship between environments and entrepreneurial teams in entrepreneurship literature.

Second, our research responds to the calls for a deeper examination of humble leader behaviors (Weick, 2001; Uhl-Bien, 2006), especially those for exploring why a humble leader would be selected or fostered in an organizational context. Although humble leader behaviors have been proved to bring many positive outcomes, people still know little about why some leaders are humbler than others. Different from the previous research focusing on personalities as antecedents of humble leadership, our research attempts to explore the contextual antecedents of humble leadership and its mechanism. By revealing dynamic work environments as the antecedent and its mechanism, this study contributed to the understanding of leader humble behaviors.

Third, although uncertainty reduction theory has been applied in several organizational researches, we are one of the first to apply the uncertainty reduction theory in the field of entrepreneurship. By using this theory, our research not only reveals why and when dynamic work environments can affect entrepreneur’s humble leader behaviors, but also broadens the uncertainty reduction theory. We prove dynamic work environment can also evoke individuals motivation toward uncertainty reduction, and we show that feedback seeking and humble leader behaviors can be useful for entrepreneurs to reduce their uncertainty.

### Limitations and Future Research Directions

There are some limitations to this paper. First, although we collected data in three waves to measure the independent variables and dependent variable separately to avoid the common method bias, our study still bears the risk of being unable to produce causal inference. To provide more evidences, longitudinal design and experimental design should be performed.

Second, as the first study on this research question, our research can only discuss one kind of mechanism that explains the relationship between dynamic work environments and entrepreneur’s humble leader behaviors. For the future studies on this topic, applying more processing variables to demonstrate the underlying mechanisms are recommended.

Furthermore, considering the potential negative effects of dynamic work environments, such as anxiety and information explosion, it’s also interesting to explore what potential negative effects dynamic environments may have on leadership behaviors. Thus, future research is expected to examine the ‘dark side’ of dynamic work environments, especially the effects on entrepreneurs.

### Practical Implication

Based on our empirical results, some important practical implications for entrepreneurial teams can be drawn. First, most entrepreneurs may treat dynamic work environment as a huge disaster to them. They are afraid of dynamic environments and exaggerate its negative effects. However, our results show that dynamic work environments can prompt humble leader behaviors in entrepreneurs, which may bring positive outcomes to entrepreneurial teams. Thus, this paper can bring entrepreneurs a new perspective to regard and react to dynamic environment. Dynamic environment can make them stay awake and keep alert, which may stimulate their positive behaviors and better performance.

Second, our study also suggests that feedback seeking can arouse entrepreneurs’ humble leader behaviors. Considering the benefits of humble leader behaviors, entrepreneurs or professional human resources training programs could train entrepreneurs to exhibit humble leader behaviors by encouraging entrepreneurs to do more feedback-seeking behavior.

Thirdly, this research reveals that the intolerance of uncertainty of entrepreneurs is important for entrepreneurs to react to the dynamic environments positively. As current environments are very dynamic, reacting well to dynamic environments is vital for the success of entrepreneurial teams. Entrepreneurs with low intolerance of uncertainty should try their best to avoid the negative effects of this trait, and those with high intolerance of uncertainty should think about it well before deciding to be an entrepreneur.

## Conclusion

Based on the uncertainty reduction theory, we found a positive relationship between dynamic work environments and entrepreneur’s humble leader behaviors. By developing and testing a moderated mediation model, we were able to show that dynamic work environments can affect entrepreneur’s humble leader behaviors through motivating entrepreneurs’ feedback-seeking behavior. And high intolerance of uncertainty of entrepreneur can weaken the effects of dynamic work environments on entrepreneur’s humble leader behaviors *via* feedback seeking. With these results, our study links the relationship between dynamic work environments and entrepreneur’s humble leader behaviors, and its mechanism and boundary condition. Our result provides empirical and practical insights into the reaction to dynamic environments in entrepreneurial teams.

## Data Availability Statement

The raw data supporting the conclusions of this article will be made available by the authors, without undue reservation, to any qualified researcher.

## Ethics Statement

The studies involving human participants were reviewed and approved by the Beijing University of Technology. The patients/participants provided their written informed consent to participate in this study.

## Author Contributions

All authors listed have made a substantial, direct and intellectual contribution to the work, and approved it for publication.

## Conflict of Interest

The authors declare that the research was conducted in the absence of any commercial or financial relationships that could be construed as a potential conflict of interest.

## Supplementary Material

TABLE S1 Entrepreneur data.
